# Amperometric Biosensor Based on Zirconium Oxide/Polyethylene Glycol/Tyrosinase Composite Film for the Detection of Phenolic Compounds

**DOI:** 10.3390/bios6030031

**Published:** 2016-06-29

**Authors:** Nor Monica Ahmad, Jaafar Abdullah, Nor Azah Yusof, Ahmad Hazri Ab Rashid, Samsulida Abd Rahman, Md. Rakibul Hasan

**Affiliations:** 1 School of Chemistry and Environment, Faculty of Applied Science, UiTM Kuala Pilah, 72 000 Negeri Sembilan, Malaysia; 2 Department of Chemistry, Faculty of Science, Universiti Putra Malaysia, 43400 Serdang, Selangor, Malaysia; jaafar@science.upm.edu.my (J.A.); azahy@science.upm.edu.my (N.A.Y.); 3 Industrial Biotechnology Research Centre, SIRIM Berhad, 1, Persiaran Dato’ Menteri, P.O. Box 7035, Section 2, 40700 Shah Alam, Selangor, Malaysia; ahazri@sirim.my (A.H.A.R.); sulida@sirim.my (S.A.R.); 4 Nanotechnology & Catalysis Research Centre, Institute of Postgraduate Studies, University of Malaya, 50603 Kuala Lumpur, Malaysia; rakibacctdu@gmail.com

**Keywords:** tyrosinase, zirconium oxide, phenol detection

## Abstract

A phenolic biosensor based on a zirconium oxide/polyethylene glycol/tyrosinase composite film for the detection of phenolic compounds has been explored. The formation of the composite film was expected via electrostatic interaction between hexacetyltrimethylammonium bromide (CTAB), polyethylene glycol (PEG), and zirconium oxide nanoparticles casted on screen printed carbon electrode (SPCE). Herein, the electrode was treated by casting hexacetyltrimethylammonium bromide on SPCE to promote a positively charged surface. Later, zirconium oxide was mixed with polyethylene glycol and the mixture was dropped cast onto the positively charged SPCE/CTAB. Tyrosinase was further immobilized onto the modified SPCE. Characterization of the prepared nanocomposite film and the modified SPCE surface was investigated by scanning electron microscopy (SEM), Electrochemical Impedance Spectroscopy (EIS), and Cyclic voltamogram (CV). The developed biosensor exhibits rapid response for less than 10 s. Two linear calibration curves towards phenol in the concentrations ranges of 0.075–10 µM and 10–55 µM with the detection limit of 0.034 µM were obtained. The biosensor shows high sensitivity and good storage stability for at least 30 days.

## 1. Introduction

Phenol formation in the aquatic environment not only comes from natural processes but also from anthropogenic processes resulting from the direct discharge from industries [1]. The main natural processes of phenol formation are the decomposition of organic matter or synthesis by fungi and plants [2]. In terms of anthropogenic processes, phenols have widely been used in industries such as the production of plastic, textile, pharmaceutical, petroleum, pesticides, herbicides, insecticides, and fungicides [3].

The undesirability of the existence of phenols in river water is due to their strong action, high degree of poisonousness to aquatic life, in addition to their unpleasant tastes and odors. Phenolic compounds are a group of polluting chemicals that are easily absorbed by animals and humans through the skin and mucous membranes. These substances are toxic to most organs and tissues, especially in the lungs, liver, kidneys, and genitourinary system [4].

Even with a lower concentration, phenols are considered to be dangerous substances and have life-threatening toxicities. Phenols have been listed as major toxic pollutants by the United States Environmental Protection Agency (EPA) and many other countries around the world [4]. The European Union has established maximum permission level of phenols in water for human consumption which is within the concentration range of 0.1 and 0.5 mg·L^−1^.

Phenolic compounds are one of the major contaminants in waters. Thus, the ability to detect its presence represents a benefit to the protection of public health and the environment. Currently, there are many methods for the detection of phenol in the environment, such as reversed phase liquid chromatography [5], spectrophotometric [2,6], and high performances liquid chromatography [7,8]. However, these methods involve expensive instrumentation, require skill personnel to handle the instrumentation, and necessitate complicated pre-processing of sample, all of which make these methods unsuitable for field monitoring [9].

In contrast, electrochemical sensors offer numerous advantages over conventional methods because of the simplicity, sensitivity, rapidity and less, if any, tedious pre-treatment for samples. The key challenges in the development of biosensors are to improve and diversify techniques for protein entrapment in order to achieve better performance and stability. In view of this challenge, there is a genuine need for new technologies, such as the utilization of nanomaterials for the improvement of the analytical performances of developed sensors. This is because most of the samples are taken from natural resources which normally contain very little amounts of phenol content; as such, a very low detection limit of the environmental biosensor will enhance the capability of the biosensor for in situ applications.

Zirconium oxide has been reported for the past few years for the detection of many analytes, such as the detection of *Escherichia coli* [10], choline [11], hydrogen peroxide [12,13,14], urea [15], and glucose [16].

The metal oxide offers good compatibility and a high isoelectric point. Thus, it provides a template for the electrostatic interaction between enzyme, polymer, and surfactant. As a result, the cross-linker, such as glutaraldehyde, could be avoided in the enzyme immobilization step. The use of glutaraldehyde at excess amounts usually will cause the conformation change of an enzyme and therefore result in the enzyme becoming denatured [16,17].

The development of phenolic biosensors based on metal oxides have been explored by many researchers; for example, a zinc oxide derived tyrosinase biosensor which has been shown to exhibit good sensitivity and a lower detection limit of 0.05 μM [18]. In considering another example, iron oxide has been applied in the development of a biosensor by incorporating a multi-walled carbon nanotube and polyaniline electrodeposited onto a gold electrode; in doing so, it was found that the biosensor showed better performance with high sensitivity and had a low detection limit of 0.03 μM [19].

**Figure 1:**
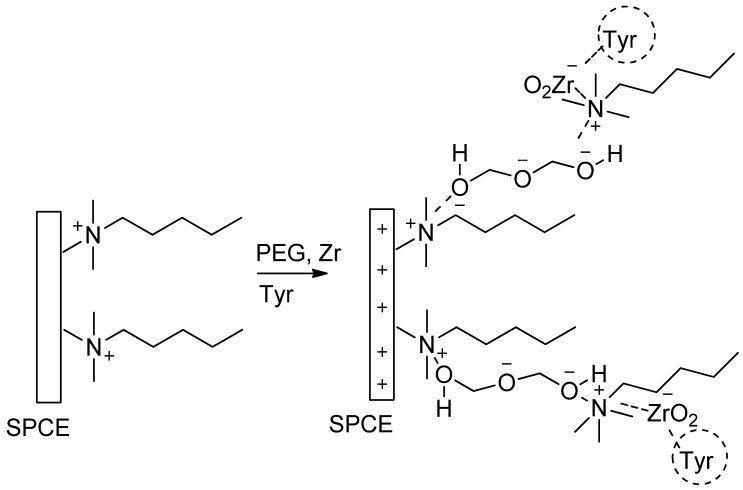
Possible assembling process of CTAB (hexacetyltrimethylammonium bromide)/PEG polyethylene glycol)-ZrO_2_/tyrosinase on screen printed carbon electrode. SPCE, screen printed carbon electrode.

Figure 1 illustrates the possible assembling process of a CTAB (hexacetyltrimethylammonium bromide)/PEG (polyethylene glycol)-ZrO_2_/tyrosinase composite on screen printed carbon electrode. Firstly, CTAB provides a positive charge on the surface of the electrode, and then it is electrostatically bound to oxygen in polyethylene glycol. At pH 6, zirconium oxide (ZrO_2_) tends to form a negative surface charge [13], which later binds to a tyrosinase enzyme. Furthermore, ZrO_2_ has an affinity towards proteins since amine and carboxyl groups in the enzyme act as a ligand to ZrO_2_ [17]. Therefore, the idea of this study is to explore the advantage of ZrO_2_ nanoparticles in combination with polyethylene glycol for the development of a phenolic biosensor which has high sensitivity, selectivity, simple technique, and fast detection.

In this research, polyethylene glycol, zirconium oxide nanoparticles, and hexacetyltrimethylammonium bromide had been used as a matrix since the synergistic effect of them in combination ensures the stability of the matrix and also will produce a phenolic biosensor with a comparable performance against the current studies. Polyethylene glycol (PEG) is a neutral non-ionic polymer with no charge on its backbone [20]; it contains an oxygen atom along its backbone, and therefore an electron pair is moving around the atom. As the electrons are moving along the atom, an electric current occurs and may improve the conductivity of the nanocomposite [21]. Herein, hexacetyltrimethylammonium bromide (CTAB) acts as a surfactant making a nonpolar chain of CTAB that interacts with the neutral polyethylene glycol.

## 2. Materials and Methods

### 2.1. Reagents

Tyrosinase from mushrooms (T3824-25KU), phenol, hexacetyltrimethylammonium bromide (CTAB), Polyethylene glycol (PEG), and zirconium oxide nanoparticles (ZrO_2_) (size less than 100 nm) were purchased from Sigma. Ascorbic acid (Unilab, Mumbai, India ), uric acid (Sigma, St. Louis, MO, USA), hydrogen peroxide (Merck, Kenilworth, NJ, USA), glucose (Univar, Downers Grove, IL, USA), magnesium sulfateheptahydrate (Fluka, St. Louis, MO, USA), calcium chloride hydrate (Univar), iron (III) chloride hexahydrate (sigma-Aldrich), *p*-cresol (Sigma), 4-chlorophenol (Sigma), and *o*-kresol (Aldrich, St. Louis, MO, USA ) were analytical grade and dissolved in phosphate buffer upon being used. All chemicals were freshly prepared prior to use. All aqueous solutions were prepared using deionized water.

### 2.2. Apparatus

Cyclic voltammetric, electrochemical impedance spectroscopy, and amperometric measurements were performed using potentiostat (Autolab) using a two electrode system. Working electrodes were modified using CTAB/PEG-ZrO_2_ with Ag/AgCl acting as a reference electrode and a Pt wire acting as the counter electrode. All electrochemical experiments were carried out in 5 mL of 0.050 mM phosphate buffer (pH 6.0). Scanning electron microscope- Energy-dispersive X-ray spectroscopy (SEM-EDX) was carried out using Carlzeiss Supra 40 VP FESEM. Fourier transform infrared spectroscopy (FTIR)analysis was conducted using Perkin Elmer Spectrum 100. 

### 2.3. Preparation of Enzyme Electrode

A solution of hexacetyltrimethyl ammonium bromide (54 mg/mL) was prepared in 0.5 M NaCl pH 8.0. CTAB solution (2.5 μL) was drop casted onto the surface of a working electrode and allowed to dry at room temperature for 1 h. Zirconium oxide powder (18 mg) was dispersed in the phosphate buffer solution (pH 8). On the other hand, 18 μL of polyethylene glycol was dissolved in 0.1 M NaCl (pH 5.4). Next, the latter two solutions were mixed at a certain ratio and pipetted on top of the modified electrode with CTAB. Then, 2.0 uL of ZrO_2_/PEG was dropped onto the electrode surface. Finally, 2.0 µL of tyrosinase (10 mg/mL) was pipetted onto the electrode and allowed to dry at 4 °C overnight.

### 2.4. Optimization and Performance Studies of the Biosensor

A series of optimization studies were carried out, including tests to determine the effect of applied potential, pH buffer, concentration of nanoparticles, and influence of the PEG-ZrO_2_ layer. The study was conducted using cyclic voltamogram and amperometric techniques. Phenol substrate was diluted from 10 µM to the final concentration using a phosphate buffer solution (pH 6). Electrochemical studies, including cyclic voltammetric, amperometric, and impedance measurements, were done using SPCE, SPCE/CTAB, SPCE/CTAB/PEG, and SPCE/CTAB/PEG-ZrO_2_.

## 3. Results and Discussion

### 3.1. Characterization of the Modified SPCE

**Figure 2:**
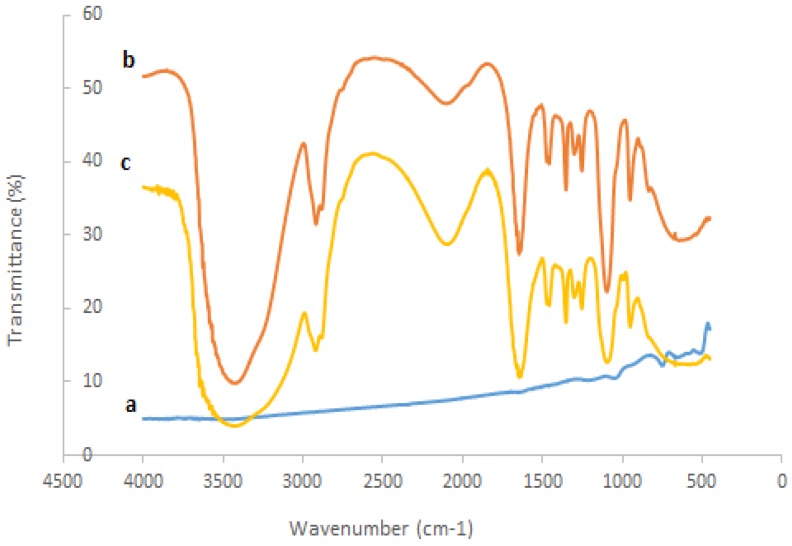
Fourier transform infrared spectroscopy spectra of (**a**) ZrO_2_; (**b**) pure tyrosinase; (**c**) PEG-ZrO_2_/tyrosinase.

The interaction between nanosized ZrO_2_ and polyethylene glycol to the tyrosinase could be studied by comparing the FTIR spectra of the ZrO_2_, pure tyrosinase, polyethylene glycol, and PEG-ZrO_2_/tyrosinase, as shown in Figure 2. As can be noted in pure tyrosinase (spectrum b), peaks from 1750 to 1600 cm^−1^, 1600 to 1500 cm^−1^, and 1350 to 1200 cm^−1^ are assigned as amide I, amide II, amide III, respectively [22]. When tyrosinase interacted with ZrO_2_ (spectrum c), the spectrum did not change dramatically, which indicates the metal oxide does not alter the native structure of the enzyme.

**Figure 3:**
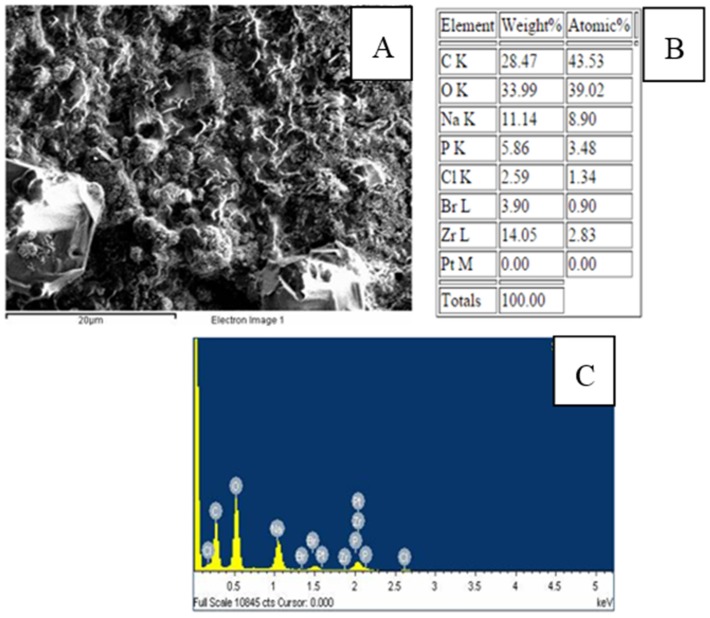
Scanning electron microscopy (SEM) photographs (**A**); table of EDX analysis; (**B**) and spectrum of SPCE/PEG-ZrO_2_ (**C**).

**Figure 4:**
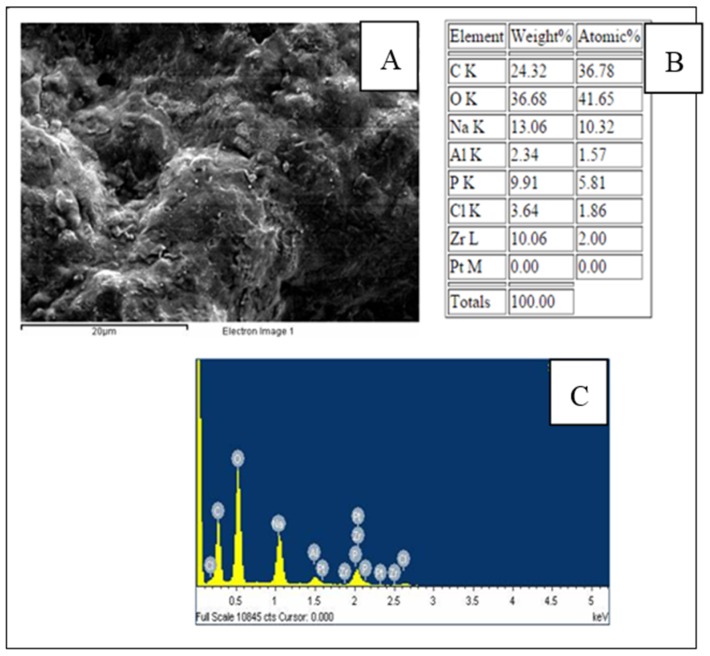
SEM photographs (**A**); EDX data; (**B**); and spectrum of SPCE/PEG-ZrO_2_/tyrosinase (**C**).

A scanning election microscopy (SEM) image of a CTAB/PEG/ZrO_2_ nanocomposite film is shown in Figure 3. It was found that the CTAB/PEG/ZrO_2_ modified electrode exhibits uniform and tiny holes over the whole surface. Energy-dispersive X-ray spectroscopy (EDX) was used to analyze the elemental composition of the modifiers on the SPCE. The spectrum (Figure 3C) shows the presence of carbon (C), oxygen (O), sodium (Na), phosphorus (P), chlorine (Cl), bromine (Br), and zirconium (Zr) on the modified electrode, which indicates the successful deposition of CTAB/PEG/ZrO_2_ on the electrode. However, when the enzyme was immobilized on the composite (Figure 4), a larger coagulant appeared, thereby showing that the pores on the surface had been totally covered by the enzyme. Data analyzed by EDX proved that the zirconium was successfully added into the nanocomposite (Figure 4B,C)

### 3.2. Electrochemical Studies

**Figure 5:**
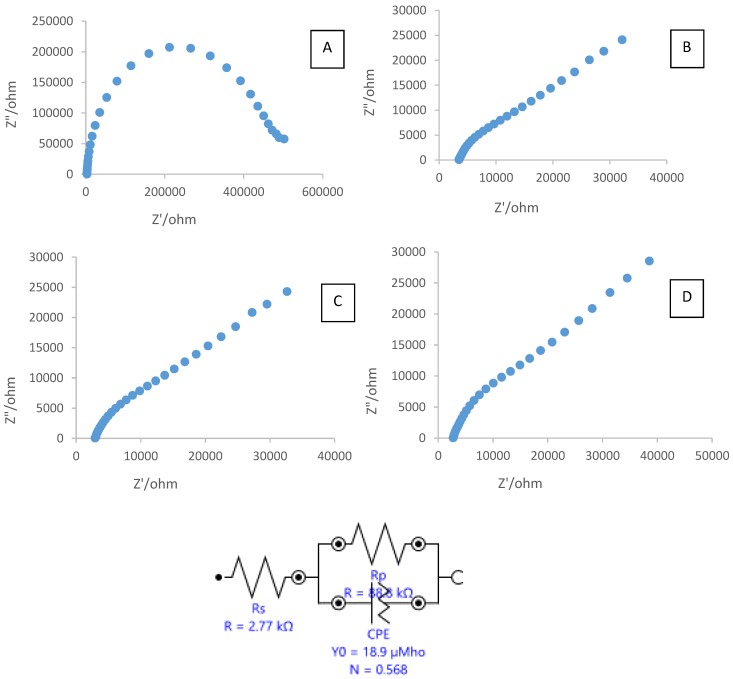
Electrochemical impedance spectroscopy (EIS) of: (**A**) bare SPCE; (**B**) SPCE/CTAB; SPCE/CTAB/PEG; (**C**) and SPCE/CTAB/PEG-ZrO_2_ (**D**) in 5.0 mM K_4_Fe(CN)_6_ containing 0.1 M KCl as a supporting electrolyte.

Electrochemical impedance spectroscopy (EIS) is a common method to investigate the interfacial properties of a modified electrode surface during the stepwise-assembly processes. Figure 5 displays the Nyquist plots of the EIS of the bare electrode (electrode A), SPCE modified CTAB (electrode B), SPCE modified CTAB/PEG (electrode C), and SPCE modified CTAB/PEG-ZrO_2_ (electrode D), respectively.

The simulated values of charge transfer resistance (R_CT_) at the bare unmodified electrode, SPCE with CTAB, SPCE casting with CTAB/PEG, and SPCE casting with CTAB/PEG-ZrO_2_ were 473,180 Ω, 99,645 Ω, 88,762 Ω, and 88,077 Ω, respectively. After modification of the SPCE with CTAB, charge transfer resistance was found to decrease, thereby showing that the conductivity has been improved, and was further decreased when PEG was added to the electrode. Upon addition of the zirconium oxide to the composite, charge transfer resistance was found to change by a slight decrease, thus showing that the material enhanced the conductivity of the modified electrode. Theoretically, ZrO_2_ nanoparticles behave as a nanoscale electrode, boosting electron conveyance between the surface of electrode and ferrocyanide ions. Q similar finding was also reported by other researchers [10] where the presence of nano-ZrO_2_ was found to increase the effective electroactive surface area, resulting in enhancement of the diffusion of redox species on the electrode surface. The value of R_CT_ shows a decreased resistance when zirconium oxide was added to the bare Indium Tin Oxide (ITO) electrode. In another study [11], the value of R_CT_ shows a decreased resistance when a zirconium oxide/multi-walled carbon nanotube was added to the glassy carbon electrode. Furthermore, Zhao et al. [13] stated that the addition of ZrO_2_ into the nonconductive chitosan decreased the resistance value, as indicated in the Nyquist plot. This behavior is due to ZrO_2_ being a semiconductor material, as such, the electron transfer from [(FeCN)_6_^3−^/^4−^] to the electrode surface becomes easier.

### 3.3. Cyclic Voltamogram

**Figure 6:**
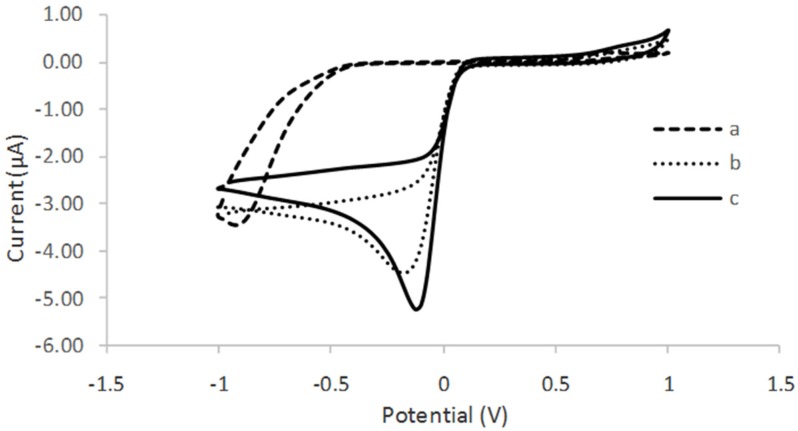
Cyclic voltamogram of (**a**) SPCE/tyrosinase; (**b**) SPCE/CTAB/PEG/tyrosinase; and (**c**) SPCE/CTAB/ZrO_2_-PEG/tyrosinase in the presence of 10 µM phenol in 50 mM phosphate buffer solution (PBS) pH 6.0.

**Figure 7:**
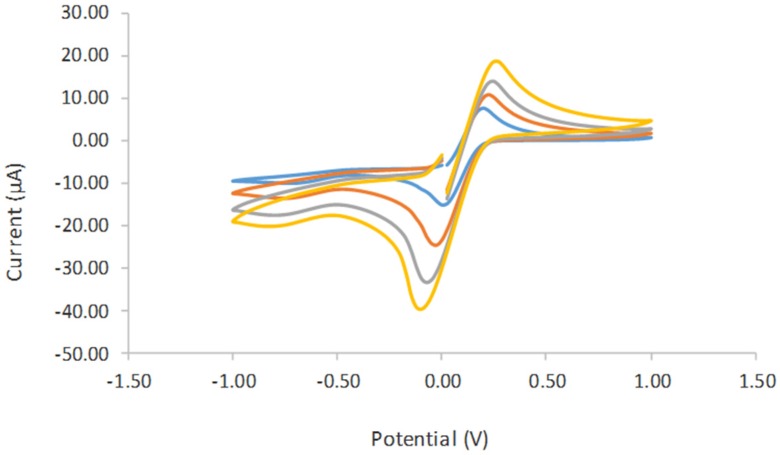
Cyclic voltamogram study on the effect of the scan rate from 0.01 V/s until 0.09 V/s in 5.0 mM K_4_Fe(CN)_6_ containing 0.1 M KCl as a supporting electrolyte. 3.5. Optimization of Experimental Parameters

The electrochemical behavior of the different modified electrodes was investigated using 0.05 M phosphate buffer solution PBS (pH 6), consisting of 0.1 M KCl at the scan rate of 0.01 V/s. Figure 6 shows the cyclic voltamogram (CV) of tyrosinase immobilized on SPCE (curve a), tyrosinase immobilized on SPCE/CTAB/PEG (curve b), and tyrosinase immobilized on SPCE/CTAB/ZrO_2_-PEG (curve c). A poor reduction peak current was observed when tyrosinase was immobilized on an unmodified electrode. A similar finding was also reported by Apetrei et al. (2015) [23]. Instead, a clear and better reduction peak current occurred at a lower potential when zirconium and polyethylene glycol were used as supporting matrix, which provided a higher surface area and good conductivity. The effect of the scan rate (Figure 7) from 10 mVs^−1^ to 80 mVs^−1^ on the biosensor response in the presence of 10 µM phenol in 0.05 M phosphate buffer solution (pH 6.0) was also investigated. The peak currents were linearly proportional to the scan rates, thereby indicating a redox property of the modified electrodes [12] and surface-controlled electrode process [14].

In this work, the electrochemical measurements have been performed using a chronoamperometric technique to observe the performance of the immobilized tyrosinase on PEG-ZrO_2_ composite film. Tyrosinase is a copper containing enzyme that catalyzes the oxidation of phenol group to *o*-quinone, which is commonly used in the detection of phenolic compounds. The oxidation of phenols by tyrosinase can be presented according to the following reactions:

Phenol + tyrosinase → catechol(1)

Catechol + tyrosinase → *o*-quinone + H_2_O (2)

*o*-quinone + H^+^ + 2e^−^ → catechol (3)

The oxidation of phenol by tyrosinase has two steps: phenol is oxidized to catechol (*o*-benzenediol), and then further oxidized by tyrosinase to *o*-quinone. The liberated quinone species can also be electrochemically reduced, and the reaction can be measured at a lower potential [24,25]. Thus, this provides the additional advantage of the enzymatic electrochemical recycling of the substrate, giving rise to signal amplification.

**Figure 8:**
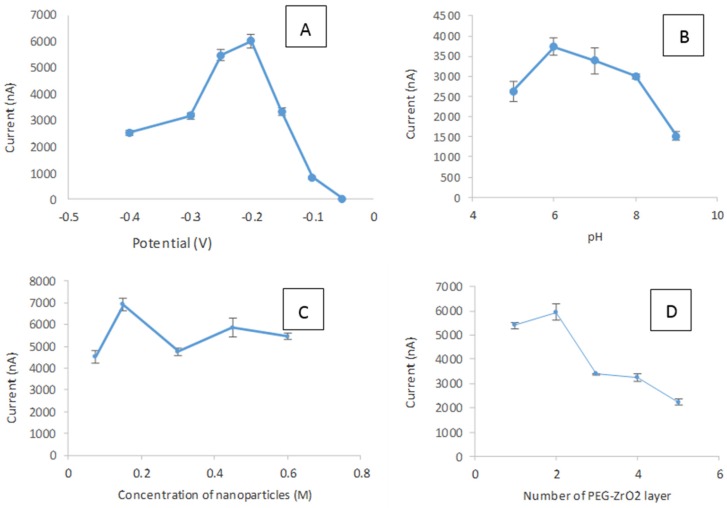
The effect of applied potential (**A**); pH buffer (**B**); concentration of nanoparticles (**C**); and number of PEG-ZrO_2_ layers on modified SPCE (**D**). Phenol concentration and applied potential were fixed, respectively, at 10 µM and −0.2 V vs. AgCl.

In this work, the effect of applied potential was investigated (Figure 8A) in the potential range of −0.05 V to −0.4 V vs. AgCl, and the optimum response of the immobilized tyrosinase on PEG-ZrO_2_ composite film was observed at the applied potential of −0.2 V vs. AgCl. Thus, the applied potential of −0.2 V was chosen for further study. The influence of pH on the biosensor response is shown in Figure 8B. The maximum current response was observed at pH 6.0. This is in accordance with the work reported by Xue et al. [26], Cheng [27], and Liu et al. [28] which indicate that the immobilization of enzymes does not drastically alter their pH response characteristics. Thus this pH was chosen for subsequent studies.

The effect of the concentration of the metal oxide incorporated into the nanocomposite film was also optimized, as shown in Figure 8C. It can be noted that the concentration of 0.075 M ZrO_2_ nanoparticles was sufficient to facilitate the electron transfer since the higher concentrations of metal oxide show reduced current signals. The effect of the film thickness of the composite film on the biosensor response was also studied in this work. Figure 8D shows the different current responses produced when a different number of layers was applied on the electrode surface. The biosensor response decreased with the increase in film thickness [29,30,31,32]. This phenomenon suggests that the thicker film that formed on the surface of the electrode caused the interfacial charge transfer between the SPCE and substrate to become difficult [9]. The enzyme has the capability to transfer electrons during oxidation and reduction when there is only a thin film on the electrode surface. With thicker PEG-ZrO_2_ films, the distance between the biomolecules and the electrodes are fully occupied with the thick matrix, and it becomes more difficult for the electron transfers to occur [20]. Therefore, by considering the activity and sensitivity of CTAB/ZrO_2_-PEG/tyrosinase towards phenol, the optimum thickness of one layer was selected for the subsequent experiment.

### 3.4. Interference Studies

The selectivity of the prepared sensor based on SPCE/CTAB/PEG-ZrO_2_/tyrosinase was also investigated by measuring the amperometric response towards 4 µM phenol in the presence of some possible interference ions. Table 1 shows the change in current response in the presence of interfering substances. It can be noted that uric acid, glucose, Mg^2+^, Ca^2+^, and Fe^3+^ did not show any significant interfering effect on the determination of phenol. However, ascorbic acid and H_2_O_2_ were shown to cause interference. This might be related to ascorbic acid being one of the enzyme inhibitors, which might reduce the tyrosinase activity.

### 3.5. Analytical Performance of the Developed Biosensor

**Figure 9:**
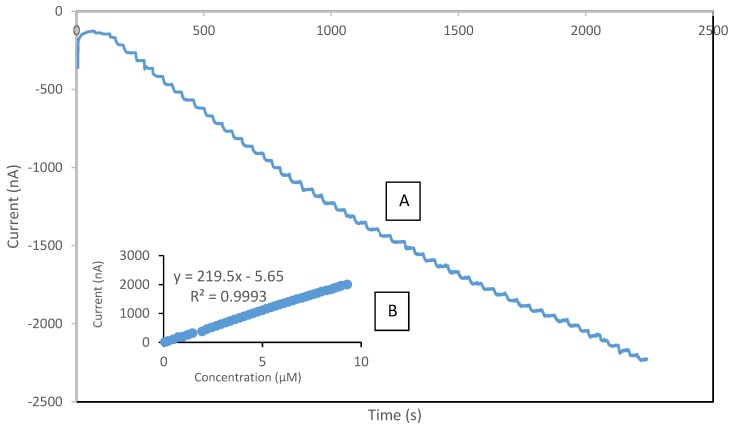
Typical current-time response curve (**A**); and calibration curves of phenol (**B**) obtained with SPCE/CTAB/PEG-ZrO_2_/Tyr on screen printed carbon electrode with the phenol concentration range of 0.075–10 µM.

**Figure 10:**
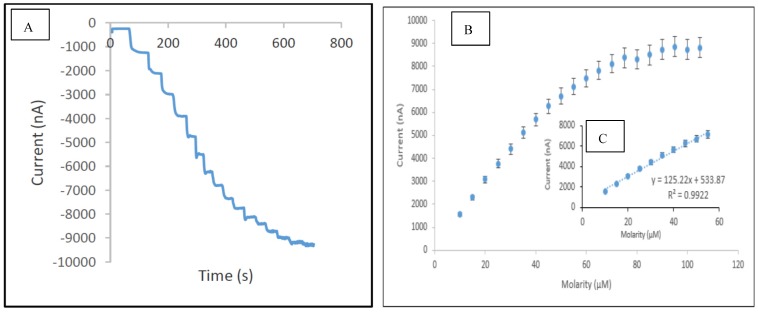
Typical current-time response curve (**A**); calibration curves for the concentration of phenol range 10–120 µM (**B**); and concentration of phenol range 10–55 µM (**C**) obtained with SPCE/CTAB/PEG-ZrO_2_/Tyr.

In this study, the performance of the developed sensor for the detection of phenol was investigated under optimum conditions in the concentration range of 0.075 µM to 120 µM. Table 2 presents the analytical characteristic of biosensors reported in literature for phenolic detection. The calibration curve was performed separately for the lower range (0.075–10 µM) and higher range (10–55 µM), respectively. It was observed that above the concentration of 55 µM, the sensor response reached the maximum value, thus indicating the saturation point. The reduction currents are linear to the concentration of phenol ranging from 0.075 to 10 µM and 10 to 55 µM with correlation coefficients 0.999 (Figure 9) and 0.992 (Figure 10), respectively, with the detection limit of 0.034 µM (S/N = 3). The sensitivity of SPCE/CTAB/PEG-ZrO_2_/tyrosinase obtained was 219.5 (mA/M), which is slightly higher than previous work using zinc oxide nanoparticles [18]. The response time of the biosensor was found to be less than 10 s. This fast response time is comparable with others studies [9,18] and might be contributed by the zirconium oxide, which promotes better conductivity of the modified electrode [10,12].

**Figure 11:**
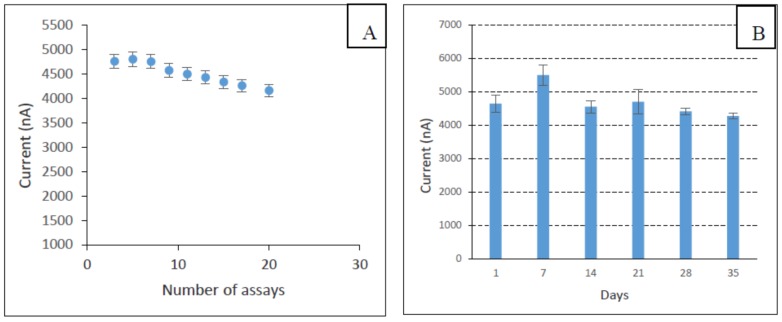
The repeatability of the developed biosensor (**A**) and storage stability of the fabricated biosensor (**B**).

The reproducibility of the biosensor fabrication was investigated by measuring the response of the sensor towards a fixed concentration of phenol (10 µM) using different electrodes. Three different enzyme electrodes were tested and the relative standard deviation (RSD) value was calculated to be 6.88%. The repeatability of the sensor was also evaluated (Figure 11A), and the RSD of 8.8% was observed for twenty successive assays of phenol (10 µM). The storage stability of the enzyme electrodes when stored at 4 °C was investigated, and it was found to be stable for at least 30 days (Figure 11B).

### 3.6. Selectivity Study

Several phenolic compounds (phenol, 4-chlorophenol, and *p*-kresol) were tested by the proposed biosensor. Table 3 presents the response characteristics of the enzyme electrode towards different phenolic compounds. The value of the apparent Michaelis-Menten constant provides the information on the affinity of an enzyme for its substrate, where a smaller value indicates a stronger substrate binding and higher catalytic activity. In this study, the apparent Michaelis-Menten constant values ($K_{M}^{app}$) for the CTAB/PEG-ZrO_2_/tyrosinase towards phenol, catechol, and 4-chlorophenol were studied and tabulated in Table 3.

The lowest $K_{M}^{app}$ value was found for *p*-cresol. This observation is slightly better than the observation from the biosensor based on polycrystalline bismuth oxide films for *p*-cresol (36 µM) [9]. The biosensor with a lower $K_{M}^{app}$ indicates that it has the capability to detect a substrate at both a very low and high concentration and has higher affinity towards the substrate, which allows it to perform a wider linear range [25]. On the other hand, the $K_{M}^{app}$ for phenol was found to be 61.42 µM, which is fairly similar to the results from the biosensor based on iron oxide-coated carbon nanotubes (58.6 µM) [27] and better than the results reported using polycrystalline bismuth oxide films (319 µM) [9].

### 3.7. Analysis of Spiked Real Water Samples

The performances of the biosensors have been tested with the analysis of spiked real samples. Water samples were taken from three different rivers in Kuala Lumpur and were brought to the laboratory without any pre-treatment. Water samples were spiked with a known concentration of phenol (0.6 µM) and measured using the developed biosensor and the standard spectrophotometric method, respectively. Table 4 presents the result of the water samples recovered. The results demonstrate both the biosensor and the spectrophotometric method. As shown in Table 4, the calculated values of |t| are less than the tabulated t for two degrees of freedom (N1 + N2 − 2) at the 95% confidence level (which is 2.920), so there is no statistical difference in the results by the two methods. This result shows that the two methods used for the determination of phenol in the spiked water samples were in good agreement and comparable.

## 4. Conclusions

This study presents a very simple and inexpensive method for phenolic determination in water samples. Despite the simplicity and common materials used, a considerably low detection limit was obtained. This method is considered to be a good platform for future modifications using other materials. In the future, the method should be modified by introducing novel materials, such as ionic liquid or quantum dots, for exploring the effects of new and different materials.

## Figures and Tables

**Table 1:** Effect of some interference species on the sensor response.

Interferent	Coexisting Level	Change of Current Response (%)
Ascorbic acid	3 µM	−5.78
Uric acid	300 µM	−0.69
H_2_O_2_	50 µM	5.18
Glucose	1000 µM	4.65
Mg^2+^	40 µM	2.88
Ca^2+^	40 µM	3.20
Fe^3+^	40 µM	1.14

**Table 2:** Analytical characteristic of biosensors reported in literature for phenolic detection.

Composite System	Linear Range (µM)	Applied Potential (V)	Detection Limit (µM)	Response Time (s)	Reference
Zinc oxide/catechol	0.15–65	−0.2	0.05	10	[18]
Iron oxide/coliform	0.01–39	−0.2	0.005	5	[27]
Bismuth oxide/catechol	0.01–8	−0.2	0.05	8	[9]
Graphene oxide-gold nanoparticle/catechol	0.083–23	−0.25	0.024	6	[33]
Iron oxide/MWCNT/PANI Au/guaicol/polyphenol	0.1-10 10–500	−0.2	0.03	3	[19]
Zirconium oxide	0.075–10 10–55	−0.2	0.034	10	This work

**Table 3:** The response characteristics of the SPCE/CTAB/PEG/tyrosinase composite film to phenolic compound.

Phenol Compound	Linear Range (µM)	Correlation Coefficient (*R^2^*)	Detection Limit (µM)	$K_{M}^{app}$ (µM)
Phenol	0.5–9.5 10.0–55.0	0.9900 0.9993	0.0379 0.1003	61.42 193.33
4-chlorophenol	1.5–7.0	0.9942	0.2372	3650.00
*p*-kresol	0.025–1.0 1.0–12.0	0.9999 0.9997	0.0175 0.5232	1.50 5.25

**Table 4:** Determination of spiked phenol in the matrix of river water (*n* = 3).

River Water	Total Phenolic Content by Present Method (µM) Mean ± SD (*n* = 3)	Total Phenolic Content by Standard Spectrophotometric Method (µM) Mean ± SD (*n* = 3)	*T*-Test Value
River 1	0.5839 ± 0.0127	0.6675 ± 0.0092	1.5500
River 2	0.5891 ± 0.0003	0.6242 ± 0.0139	1.2448
River 3	0.5897 ± 0.0002	0.5833 ± 0.0416	0.0771

Note: The critical value, t_4_ = 2.92 (*p* = 0.05).

## Abbreviations

The following abbreviations are used in this manuscript:

MDPI	Multidisciplinary Digital Publishing Institute
DOAJ	Directory of open access journals
TLA	Three letter acronym
LD	linear dichroism
